# The Relation Between Steroid Secretion Patterns and the Androgen Receptor Gene Polymorphism on Physical Health and Psychological Well-Being—Longitudinal Findings From the Men’s Health 40+ Study

**DOI:** 10.3389/fnhum.2020.00043

**Published:** 2020-02-14

**Authors:** Tim Jonas Lacker, Andreas Walther, Serena Fiacco, Ulrike Ehlert

**Affiliations:** ^1^Clinical Psychology and Psychotherapy, University of Zurich, Zurich, Switzerland; ^2^University Research Priority Program (URPP) Dynamics of Healthy Aging, University of Zurich, Zurich, Switzerland; ^3^Biopsychology, TU Dresden, Dresden, Germany

**Keywords:** healthy aging, men’s health, androgen receptor, steroid hormones, gerontopsychology, physical health, mental health, biopsychosocial

## Abstract

Research is increasingly focusing on promoting healthy aging and the related extension of the health span by targeting crucial biological processes responsible for age-related conditions. While age-related gradual changes in steroid hormones such as testosterone, estradiol, or cortisol are well described in men, their interactions among each other or with genetic markers have not been sufficiently investigated with regard to physical health or psychological well-being. More specifically, the examination of age-related alterations in hormone interactions and the androgen receptor polymorphism, which modulates androgen action on target cells, in relation to physical health and psychological well-being represents a promising avenue for research on healthy aging in men. A total of 97 healthy aging men provided complete data on psychometric health measures as well as hormonal and genetic parameters at baseline and a 4-year follow-up assessment. Fasting saliva samples were taken at 8:00 am under standardized laboratory conditions, while the androgen receptor gene polymorphism was analyzed from dried blood spots. Longitudinal analyses revealed that psychological well-being and physical health remained stable over time. Analyses indicated that E2 moderated the course of psychological well-being, while the androgen receptor gene polymorphism moderated the course of physical health. Further, T was a strong predictor of physical health. These results suggest that the hypothalamic-pituitary-gonadal (HPG) axis might be important for the maintenance of psychological well-being in men, while physical health depends more on interindividual differences in the androgen receptor gene and T.

## Introduction

Research is increasingly focusing on promoting healthy aging and the associated extension of the health span by targeting crucial biological processes responsible for age-related health conditions (Barzilai et al., 2018). Healthy aging has become one of the leading health care goals of our time, with the World Health Organization (WHO) recently declaring a “Decade of Action on Healthy Aging” (World Health Organization, 2017). Generally speaking, healthy aging can be defined as the “process of developing and maintaining the functional ability that enables well-being in older age” (World Health Organization, 2015b, p. 28). Overall, life expectancy has increased dramatically over the last 150 years, rising even more sharply in the last few decades (World Health Organization, 2015a). At the same time, due to a slower increase in birth rates, the world population is facing a growing proportion of older individuals (Beard et al., 2016). Living the gained years of life in good health is a prioritized aim not only for each individual, but also for society in general, in order to reduce the large economic burden of chronic diseases in the elderly (Beard and Bloom, 2015; World Health Organization, 2017). To achieve this, it is crucial to investigate biological influences (e.g., hormone secretion and genetic markers) on physical health and well-being, comprising both physical and psychological aspects.

Psychological well-being changes throughout the lifespan. It is mostly measured based on the individual’s subjective perception and comprises three essential components: life satisfaction, presence of positive affect and absence of negative affect (Diener and Lucas, 1999). Subjective well-being has been found to be relatively stable until very old age, whereupon it declines due to health constraints. This stability despite age-related physical health decline has been called the well-being paradox (Kunzmann et al., 2000; Hansen and Slagsvold, 2012). The individual psychological well-being can be influenced by various biological factors such as hormone secretion, with the mood-altering properties of testosterone (T) being especially crucial for aging males (Walther and Ehlert, 2015). Furthermore, genetics plays an important role, not only in physiology but also in subjective well-being (Weiss et al., 2008). More specifically, the androgen receptor gene polymorphism has been linked to personality factors that are closely associated with well-being (Weiss et al., 2008; Westberg et al., 2009). While another study with three distinct samples did not replicate the finding that the androgen receptor gene polymorphism functions as a predictor of well-being in the general population sample, it was a predictor in the two patient samples (Schneider et al., 2010). However, the aforementioned study assessed general well-being rather than specifically psychological well-being.

In most individuals, physical health deteriorates with higher age and often results in frailty and multimorbidity (Huisman et al., 2011; Lowsky et al., 2013; Guthrie and Boyd, 2018; Rockwood, 2019). However, a general population study found that even in a group of 8,059 individuals aged 85 years and older, 56% of participants reported that they did not experience any health-related limitations (Lowsky et al., 2013). Thus, one important marker of healthy aging is the subjective physical health status (Han et al., 2015). Previous research has described multiple potential biological influences, such as age-related changes in hormone secretion patterns or genetic markers (Feldman et al., 2002; Tirabassi et al., 2015; Walther et al., 2016).

Steroid hormones show distinct trajectories of stability, decrease or increase throughout the aging process. With regard to the hypothalamic-pituitary-gonadal (HPG) axis, the primary male sex steroid T significantly decreases with age, whereas estradiol (E2) shows stability or a slight decline (Vermeulen et al., 2002; Fiacco et al., 2018). For example, the effect size of the T decrease has been shown to be *r* = 0.46 in an influential population-based study with 1156 individuals (Feldman et al., 2002). These changes can mainly be attributed to the decreased function or attrition of the Leydig cells in the testes. In addition, increased concentrations of circulating sex hormone-binding globulin (SHBG) and a higher conversion rate of T to E2 by the enzyme aromatase within the body’s fat cells further lead to markedly reduced testosterone levels (Vermeulen, 2000; Samaras et al., 2012). The hypothalamic-pituitary-adrenal (HPA) axis also undergoes distinct changes throughout the aging process, leading to increased cortisol secretion (Karlamangla et al., 2013). The increase in C is a consequence of age-related changes in glucocorticoid and mineralocorticoid receptor quantities, increased corticotropin-releasing hormone secretion from the hypothalamus and decreased sensitivity of the feedback mechanisms of the HPA axis (Zhou and Swaab, 1999; Gupta and Morley, 2014). Following the principle of homeostasis (Cannon, 1929; Joseph and Whirledge, 2017), mutual adaptations between the HPA and HPG axes aiming for an endocrine balance, and also within the HPG axis itself, have been suggested as important factors determining an individual’s health (Sollberger and Ehlert, 2016). Thus, it is important to respect the interaction between the different hormones of the two main endocrine axes.

Investigating the interaction between different steroid hormones can be a promising and insightful strategy to investigate the interplay of different hormonal axes and the interplay of hormonal end products within a single endocrine axis (Sollberger and Ehlert, 2016). A prospective cohort study on over 2,500 men, investigating the hormone ratio and coronary heart disease, found positive associations of the T and C ratio with the risk of cardiovascular events (Smith et al., 2005). The same study further linked the T and C ratio to physical health factors such as BMI or blood pressure. Moreover, emotion regulation, which is closely related to psychological well-being, was positively associated with the T and C ratio in 19 participants of an fMRI study in healthy men (Denson et al., 2013). For another prominent hormone ratio, the T and E2 ratio (TE2r), results indicated a positive association with disease risk (Schneider et al., 2010; Gong et al., 2013). To the best of our knowledge, there are not yet any studies investigating the TE2r with respect to psychological well-being. Since hormone ratios can be criticized, obscuring the influence of each hormone on the effect, it is important to additionally respect literature on hormone interactions without the calculation of ratios (Sollberger and Ehlert, 2016). In a recent review, T has been described as influencing physical health factors such as frailty in elderly men (Gordon and Hubbard, 2018). T has also been found to have a positive influence on psychological well-being, while a rather low C secretion can have the same effect. Studies investigating E2 need to be done, yet (Rector and Friedman, 2018). In general, since the field of positive psychology is very young, studies specifically investigating the association between hormones and psychological well-being or their interaction are very scarce (Rector and Friedman, 2018).

Genetic differences in the androgen receptor gene influence androgen action on target cells and modulate the association between androgens and physical health and well-being. The androgen receptor gene is located on the X chromosome at the location q11-q12 and comprises eight exons. The first exon of the androgen receptor gene contains a distinct cytosine-adenine-guanine (CAG) repeat sequence, which varies in repeat length between 10 and 36 for healthy individuals (Tirabassi et al., 2015). This variation has been suggested to modulate the androgen receptor binding affinity, affecting the androgen receptor action in response to receptor binding (Chamberlain et al., 1994; Rajender et al., 2008). This could lead to altered availability and function of androgens and, since the endocrine system is dynamic in nature, the HPA axis is influenced in its’ function by this as well (Terburg et al., 2009). Previous research on the CAG repeat length and endocrine secretion has yielded mixed results, with one population-based longitudinal study reporting negative associations with T levels (Krithivas et al., 1999) and a more recent study failing to confirm this association (Eendebak et al., 2016). Another study suggested that higher CAG repeat length is not associated with altered T secretion, but is associated with increased E2 action (Huhtaniemi et al., 2009). Furthermore, associations with psychological traits have been found. More specifically, the CAG repeat length was negatively associated with aggression (*r* = −0.365) in a study of 645 convicted criminals, showing a moderate effect size (Rajender et al., 2008). Furthermore, individuals in the shorter CAG repeats group showed higher values on extraversion and neuroticism in a study of 141 individuals (Westberg et al., 2009). All of the above-mentioned traits can influence psychological well-being (Strickhouser et al., 2017). Finally, the CAG repeat length has previously been associated with health conditions. A low CAG repeat was associated with a greater risk of prostate cancer, with odds ratios of up to 3.7, in an influential review article (Nelson and Witte, 2002), while a higher CAG repeat was associated with infertility in a study comparing 37 infertile with 50 fertile individuals (Wallerand et al., 2001).

The biopsychosocial model (Engel, 1980; Wade and Halligan, 2017) postulates interactions between psychosocial and biological markers. However, to date, no study has investigated the interplay between the influence of hormonal axes and the androgen receptor gene on changes in psychological well-being and subjective physical health specifically in healthy aging men. We hypothesize that physical health decreases, psychological well-being remains stable and both, the TCr and TE2r decrease. Furthermore, we hypothesize that the interactions between the assessed steroid hormones, through their rather short-term secretory nature, influence the change in psychological well-being, while the CAG repeat length moderates the change in physical function.

## Materials and Methods

### Participants

For the current study, we longitudinally investigated 97 healthy aging men from the Men’s Health 40+ study at two timepoints (Walther et al., 2016). All men were between 40 and 75 years of age at the time of the baseline measurement. To ensure the investigation of healthy aging men at both time points, participants were screened before inclusion in the study. First, all participants were asked “How would you describe your current health?,” which is the first item of the Short Form 36 Health Survey (Bullinger and Kirchberger, 1998). This item is rated on a scale encompassing very bad, bad, good, very good and excellent. To be eligible for study inclusion, participants had to be fluent in the German language and had to describe their health as at least good at both time points, suggesting that participants perceived a continuously high level of subjective health during the time of study. Participants who reported acute or chronic medical conditions, psychiatric disorders, psychopharmacological treatment (including T supplementation) or psychotherapy were excluded. The study was approved by the local ethics committee and all participants accepted the terms of the study and gave their written informed consent.

### Procedure

The study comprised two timepoints spanning 4 years. At each time point, the study was divided into two parts. The first part consisted of three consecutive questionnaire batteries, each lasting for 1 h, including questionnaires measuring subjective health and physical health. For the second part of the study, participants underwent a laboratory assessment at the laboratory facilities of the psychological institute. During this assessment, participants provided saliva samples and blood samples and underwent various other physiological measures such as bioelectrical impedance analysis to investigate the body composition. Detailed information on the study procedure is reported elsewhere (Walther et al., 2016).

### Questionnaire

For the investigation of psychological well-being and physical health, the German version of the Short Form 36 Health Survey (SF-36) questionnaire was used (Bullinger and Kirchberger, 1998). The SF-36 is a widely used, well-validated self-report questionnaire consisting of eight subscales, of which the psychological well-being and physical health subscales were used for the current study. The internal consistency for the scales used was in line with the originally reported values, being mostly over *α* = 0.70. For the physical health scale, the internal consistency was *α* = 0.66 at baseline and *α* = 0.70 at follow-up. For the psychological well-being scale, the internal consistency was *α* = 0.80 at baseline and *α* = 0.73 at follow-up.

### Hormone Analyses

All hormone samples were taken between 8:00 and 8:15 a.m. during the laboratory assessment. Participants provided fasting saliva samples (SaliCaps, IBL International GmbH, Hamburg, Germany) under the supervision of trained study personnel. After collection, samples were stored at −20°C until analysis. T and E2 were analyzed using a luminescence immunoassay (Goncharov et al., 2006). The intra- and inter-assay variation for T and E2 was below 10%, with sensitivities of 1.8 pg/ml for T and 0.3 pg/ml for E2. C was measured using an enzyme-linked immunosorbent assay, with an intra- and inter-assay variation below 10% and a sensitivity of 30 pg/ml (Chiappin et al., 2007). To standardize the units, C was transformed from nmol/L to pg/ml. Afterward, hormone ratios were calculated. After calculation, both ratios were log-transformed to smooth the distribution and therefore were then normally distributed (Sollberger and Ehlert, 2016).

### Genetic Analyses

Blood samples were assessed using the dried blood spot method (Fischer et al., 2019b), in which participants provided four drops of blood on a filter paper consisting of pure cellulose (Protein Saver Snap Apart, Forest Farm Industrial Estate, Cardiff, UK). Analysis of DNA with dried blood spots has been shown to be a reliable and valid procedure to analyze polymorphisms (Demirev, 2013). The sample collection took place under standardized laboratory conditions immediately after saliva samples were taken. After drying the samples for at least 4 h, they were frozen and stored at −20°C until analysis. During analysis, genomic DNA was extracted from three filter paper punches of 3 mm diameter each, using the QIAGEN QIAamp DNA Investigator Kit (Qiagen, Hombrechtikon, Switzerland). The genetic analyses themselves were conducted applying the capillary electrophoresis method, using the Applied Biosystems 3730XL Sequencer (Thermo Fisher Scientific, Waltham, MA, USA) with protocols and primers according to Westberg et al. (2001). For the determination of the fragment lengths, the GeneMapper Software v3.7 (Thermo Fisher Scientific, Waltham, MA, USA) was used.

### Statistical Analysis

To investigate the association of steroid hormone and their interactions and the androgen receptor gene polymorphism with the change in psychological well-being and physical health over time, two different sets of analyses were conducted. All analyses were performed in R (v 3.4.3), using the “lme4” package (Bates et al., 2015). The normality of the residuals was tested visually by inspecting the QQ-plots. Hormone variables were log-transformed to facilitate normality of the residuals. No crude violations of the normality of the residuals were detected. The first step of the analyses investigated the longitudinal linear mixed model change in psychological well-being, self-reported physical health and the hormone ratios. In a subsequent step, two separate moderation analyses were conducted to investigate whether the hormones and their interactions or the androgen receptor gene polymorphism influence the change in psychological well-being or physical health over time. It is important to note that for all analyses with dynamic parameters (except for the androgen receptor), we used longitudinal data instead of only baseline values, in order to incorporate the dynamic nature into the analyses. Moderation analyses with both hormone ratios were conducted in individual models for each ratio, with subsequent individual interaction analyses of the single hormones. Models for the hormonal interactions included both interaction terms for T and E2, or T and C, respectively, in the same model. The two-step analysis was conducted to enable a broader analysis of steroid hormone interactions, followed by a detailed, statistical interaction term analysis to determine which of the hormones exhibits a stronger impact on the effect. Taken together there were seven separate models. Two individual models for the hormone ratios*time, three separate models for all single hormones as predictors and one model for the T*time and C*time and one for the T*time and E2*time interaction terms. All analyses were controlled for physical activity, educational level, income, medication intake, and fat mass. To control for differing trajectories between different age groups, age was also used as a covariate. Furthermore, for all analyses with the hormones and their interactions, the androgen receptor gene polymorphism was added as a covariate. For the androgen receptor gene polymorphism moderations (i.e., CAG repeat length), (longitudinal) *T* values were added as a covariate to account for the potential interdependence of the androgen receptor and T.

## Results

### Sample Characteristics

At baseline, 271 participants were included in the study. However, only 130 participants completed the screening for the follow-up and 97 participants completed the whole study at the second time point. The participants were invited at the T1 for a cross-sectional study and were then re-invited for a follow-up approximately 4 years after initial testing. Due to the initial cross-sectional design of the study, most participants who refused participation for the second wave did so because they did not expect a follow-up. After completion of the screening, participants who dropped out reported that they did not have sufficient time for the study or they were no longer eligible (i.e., no longer reported at least good self-rated health). Detailed information on the dropout rate is described elsewhere (Lacker et al., 2019).

The sample characteristics are described in Table 1. Participants were on average 61.26 (*SD* = 10.02) years old at baseline and 65.18 (*SD* = 9.98) years old at follow-up. The BMI was approximately 25 at both time points, with only minor changes. The majority of participants were in a relationship or married at both time points. Over 40% of participants held a university degree, while 24% of participants reported having completed secondary school as their highest educational attainment. Most participants earned between 50,000 and 1,50,000 CHF (50,000–1,50,000 USD) annually. At both time points, over 80% of participants reported being non-smokers. Around 60% of participants at baseline and 53% at follow-up did not take any regular medication. Of the participants taking medication, almost half took antihypertensive medication or vitamins and minerals, and a further 50% took “other” medication, which included mostly cholesterol and bowel-regulating medications. With regard to physical activity, baseline results indicated that 66% of participants were physically active for 4–6 h or more per week, while this proportion increased by 12%, to a total of 78%, at follow-up.

**Table 1:** Sample characteristics.

	Baseline			Follow-Up		
	*N*	M/Freq.	*SD*/%	*N*	M/Freq.	*SD*/%
Age	97	61.26	10.02	97	65.18	9.98
BMI	95	25.16	2.57	97	25.51	4.31
Fat mass in percent (%)	95	22.42	5.43	97	20.58	7.03
CAG repeats	−	−	−	94	17.8	3.07
C (pg/ml)	95	1,424.3	1,396.5	97	2,169.9	1,597.5
T (pg/ml)	93	42.53	18.44	96	37.88	26.28
E2 (pg/ml)	94	1.46	1.06	97	1.63	1.69
Physical health	97	65.47	3.93	96	65.36	4.09
Psychological well-being	97	25.58	2.87	96	25.67	2.87
Marital status (%)	97			96		
Single		8	8.25		5	5.21
In relationship		8	8.25		10	10.42
Married		70	72.16		70	72.92
Divorced		10	10.31		10	10.42
Widowed		1	1.03		1	1.04
Education (%)	97			96		
Middle school		8	8.25		9	9.38
Secondary school		23	23.7		23	23.96
Grammar school		7	7.22		7	7.29
University degree		43	44.33		46	47.92
other		16	16.49		11	11.46
Income (in CHF, %)	97			96		
No income		1	1.03		1	1.04
Up to 30,000		3	3.09		3	3.13
30,001–50,000		7	7.22		4	4.17
50,001–1,00,000		35	36.08		38	39.58
1,00,001–1,50,000		35	36.08		38	39.58
1,50,001–2,00,000		12	12.37		7	7.29
More than 2,00,000		4	4.12		5	5.21
Smoking (%)	97			96		
Non-smoking		82	84.54		83	86.46
Smoking		15	15.46		13	13.54
Medication (%)	97			96		
No		59	60.08		51	53.13
Yes (details see below)		38	39.18		45	46.88
Type of medication^a^ (%)	38			45		
Antihypertensives		23	45.09		28	45.16
Vitamins and minerals		1	1.96		5	8.06
Other		27	52.94		29	46.77
Physical activity (%)	97			96		
Less than 1 h		1	1.03		1	1.04
1–3 h		32	32.99		20	20.83
4–6 h		40	41.24		51	53.13
7 or more		24	24.74		24	25.00

*^a^Participants could indicate more than one medication. Note. N, Number of participants; M, Mean; Freq., Frequency; SD, Standard deviation; %, Percent; C, Cortisol; T, Testosterone; E2, Estradiol; CHF, Swiss Francs*.

### Mixed Model Analyses and Descriptive Data

The mean CAG repeat length was 17.8 (*SD* = 3.07) and approximately normally distributed (data not shown). Participants had a raw mean C level of 1,424.3 (*SD* = 1,396.5) pg/ml at baseline, which increased to 2169.9 (*SD* = 1,597.5) pg/ml at follow-up. For raw T, the results indicated a mean T level of 42.53 (*SD* = 18.44) pg/ml at baseline and a decreased T level of 37.88 (*SD* = 26.28) at follow-up. Raw E2, however, remained rather stable, with a slight increase from 1.46 (*SD* = 1.06) pg/ml at baseline to 1.63 (*SD* = 0.17) at follow-up. For the hormone ratios, the raw Testosterone/Cortisol ratio decreased from 0.06 (*SD* = 0.05) pg/ml to 0.03 (*SD* = 0.03) pg/ml, while the raw Testosterone/Estradiol ratio decreased from 43.35 (*SD* = 30.02) pg/ml to 38.52 (*SD* = 41.95) pg/ml. Mixed model analyses for the change in single hormones can be derived from Lacker et al. (2019). The self-reported physical health remained stable, with values of 65.47 (*SD* = 3.93) at baseline and 65.36 (*SD* = 4.09) at follow-up. Psychological well-being remained similarly stable, with a mean of 25.58 (*SD* = 2.87) at baseline and 25.67 (*SD* = 2.87) at follow-up. The mixed model analyses with random intercepts (detailed information shown in Table 2) yielded no significant results for the change in physical health and psychological well-being, while both hormone ratios decreased significantly (TCr: *b* = −0.756, *SE* = 0.134, *p* < 0.001; TE2r: *b* = −0.347, *SE* = 0.098, *p* < 0.001).

**Table 2:** Results of the linear mixed model analyses.

	Linear mixed model results over time						
	AIC_0_	AIC	Estimate	*SE*	*p*	95% CI_lower_	95% CI_upper_
Physical health	1,042	1,059	0.143	0.389	0.367	−0.627	0.911
Psychological well-being	898	912	−0.058	0.262	0.825	−0.578	0.459
**Linear mixed model results moderation analyses for physical health**							
TCr*time	1,042	993	0.556	0.496	0.265	−0.436	1.534
TE2r*time	1,042	996	−0.346	0.564	0.540	−1.470	0.765
T	1,042	992	1.343	0.400	<0.001	<0.544	2.150
C	1,042	1,000	0.443	0.320	0.168	−0.194	1.093
T*time	1,042	986	−0.188	0.865	0.828	−1.960	1.530
C*time	1,042	986	0.001	0.591	0.999	−1.169	1.196
E2	1,042	1,011	0.034	0.356	0.923	−0.673	0.744
T*time	1,042	993	−1.049	0.961	0.277	−3.027	0.865
E2*time	1,042	993	0.741	0.595	0.215	−0.432	1.944
CAG repeat*time	1,042	1,010	−0.271	0.117	0.023	−0.503	−0.039
**Linear mixed model results moderation analyses for psychological well-being**							
TCr*time	898	863	−0.289	0.343	0.401	−0.974	0.387
TE2r*time	898	864	1.076	0.375	0.005	0.336	1.820
T	898	870	0.222	0.284	0.436	−0.365	0.826
C	898	870	−0.121	0.218	0.579	−0.554	0.320
T*time	898	866	−0.027	0.600	0.964	−1.238	1.157
C*time	898	866	0.533	0.411	0.198	−0.285	1.379
E2	898	876	0.414	0.246	0.094	−0.080	0.912
T*time	898	867	1.165	0.643	0.073	−0.124	2.434
E2*time	898	867	−1.006	0.395	0.013	−1.785	−0.217
CAG repeat*time	898	881	−0.081	0.079	0.312	−0.238	0.077

*N, 97; T, Testosterone; C, Cortisol; E2, Estradiol; AIC0, Akaike criteron of the null model; AIC, Akaike criterion; SE, standard error; p, *p*-value; CI, Confidence interval*.

The results of the moderation analyses are described in Table 2. Moderation analyses for the longitudinal change in physical health did not yield significant results for the interaction of T and E2, T and C and the hormone ratios. However, T was a significant predictor of physical health, meaning that it significantly influenced the intercept for physical health (*b* = 1.34, *SE* = 0.40, *p* < 0.001). Additionally, when using the CAG repeat length (*b* = −0.27, *SE* = 0.12, *p* = 0.023) as moderator (CAG repeat*time) a significant moderation effect on the change in physical health was found, showing an increase for lower CAG repeats. For the longitudinal change in psychological well-being, on the other hand, the results indicated a significant moderation effect of E2 (*b* = −1.01, *SE* = 0.40, *p* = 0.013), but only a trend for T (*b* = 1.17, *SE* = 0.64, *p* = 0.073), on the changes over time, whereas the CAG repeat and the interaction of T and C were not significant moderators of the change in psychological well-being. Furthermore, there was a significant moderation effect of the TE2r on psychological well-being (*b* = 1.076, *SE* = 0.374, *p* = 0.005).

**Figure 1:**
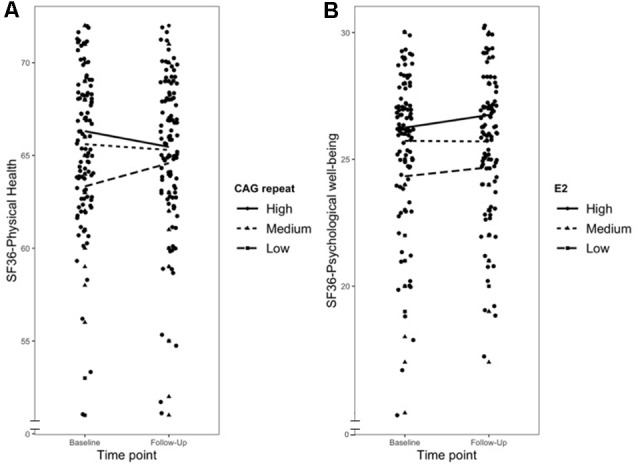
Graph showing moderation effects for all significant interactions. Panel **(A)** showing the influence of the androgen receptor polymorphism cytosine-adenine-guanine (CAG repeat) on the course of physical health, Panel **(B)** showing the influence of E2 on the course of psychological well-being. High is 1SD above the sample mean, Medium the sample mean and Low is 1SD below the sample mean.

Figure 1 graphically depicts the three significant moderation effects; however, it is important to note that the grouping was conducted for reasons of simplicity, and all moderation analyses used continuous variables as moderators.

Additionally, the supplementary material shows a correlation matrix for all variables that were used in the study. Supplementary Tables S1, S2 show bootstrapped zero-order two-tailed correlations and two-tailed partial correlations, controlling for age, education, income, fat mass, and physical activity.

## Discussion

In this longitudinal study on healthy aging men, we found that both hormone ratios decreased over time, while self-reported physical health and psychological well-being remained stable. Moreover, E2 moderated the course of psychological well-being, insofar as the change in psychological well-being differs between individuals in relation to the (longitudinal) E2 values, if *T* values are taken into account. None of the other assessed hormones moderated psychological well-being or physical health. T was, however, found to be a significant predictor of physical health. Additionally, the androgen receptor gene polymorphism had an impact on the course of physical health: the trajectory of physical health changes with the number of CAG repeats.

In general, the literature on age-related hormone alterations in men reports a decrease in sex hormones, with the exception of E2, and an increase in glucocorticoid levels is also well documented (Fiacco et al., 2018). Our findings support this, showing a decrease in T, an increase in C, and stable E2 levels (Lacker et al., 2019).

The finding that physical health and psychological well-being remained stable in our sample is highly interesting considering that in the general population, a continuous age-related decline in both health dimensions has been reported (Murphy et al., 2017). A multi-national study conducted over a similar time span in a large general population sample revealed a stronger decrease in physical health compared to our sample, but an equally low decrease in psychological well-being (Bourassa et al., 2017). The findings from another study suggest that psychological well-being, and especially dimensions such as life satisfaction or negative affect, might decline in advanced age only (Hansen and Slagsvold, 2012). However, with the present sample of middle-aged and older men, we cannot draw definitive conclusions in this regard.

E2 seems to positively influence the course of psychological well-being in specifically healthy aging men, as shown in the present analyses. Interestingly, while a recent meta-analysis showed increased E2 levels for depressed men (Fischer et al., 2019a), previous research suggested that E2 alone did not have an impact on markers of well-being such as mood (Beer et al., 2006). The latter finding was supported in our result, where the interaction of E2 was only significant after adding the interaction with T. Also in line with the latter finding, the TE2r was found to be associated with depressive symptoms in a very specific population of obese men (Monteagudo et al., 2016). The high-fat mass in these men presumably led to high aromatase activity and consequently to a parallel increase in E2 and a decrease in T (Vermeulen, 2000; Samaras et al., 2012). Concluding from these findings in obese men, we argue that it is generally not higher E2 alone that is responsible for reduced psychological well-being in aging men, but rather the interaction with reduced T levels. This ultimately results in an imbalance in the endpoints of the HPG axis, with effects on psychological well-being, where E2 is the main reason for this effect. This aspect also enables the integration with the recent meta-analysis by Fischer et al. (2019a).

The balance between the hormonal endpoints of the HPG and HPA axes may have an influence on psychological well-being. Previous research suggested functional connectivity between these two axes, through the existence of sex and adrenal steroid receptors in brain regions such as the hippocampus, amygdala or nuclei of the hypothalamus (Viau, 2002). In accordance with this, meta-analyses found T and C to be associated with mental health, which is closely related to psychological well-being (Adam et al., 2017; Walther et al., 2019). This implies that the balance between the two can also play a role in mental health maintenance. At first glance, it is thus surprising that we were unable to replicate this effect in the current sample. However, the present finding is in line with a previous report from our group in the same sample, in which we showed that psychosocial markers and biological markers change independently of each other (Lacker et al., 2019). Therefore, the lack of a relationship between the interaction of T and C and psychological well-being might be a phenomenon of healthy aging.

While the interplay between the endpoints of the HPG and HPA axes does not seem to explain changes in psychological well-being, T alone seems to be a predictor of physical health. This finding is in line with the literature. For example, a previous study with 407 participants revealed a low TCr to be an indicator of poor physical health, where T, but not C, differed significantly between a healthy and a non-healthy group (Wang et al., 2013). A recent review also supports the impact of T on physical health (Fiacco et al., 2018). In general, T shows anabolic effects, while C exhibits catabolic effects, with the former improving aspects of physical health (e.g., bone density) and the latter decreasing physical health (Dennison et al., 1999; Snyder et al., 2017). This might explain why T, but not C, was a significant predictor of physical health in our healthy aging sample. Similarly to psychological well-being, the lack of influence of the T and C interaction on physical health might reflect a healthy aging phenomenon, due to increased physical health in the investigated sample and thus adequate T and C values.

The interaction between the endpoints of the HPG axis does not seem to influence physical health in healthy aging men, as the T and E2 interactions did not have an impact on physical health in our sample. A previous general population study, however, concluded that the TE2r can be important for longevity (Menke et al., 2010). More specifically, low E2, as well as low T secretion, were associated with a higher cardiovascular risk. The same was found in another study for all-cause mortality (Hsu et al., 2016). In our study, T was a predictor of physical health only when the interaction with E2 was not included in the model. However, participants with higher T exhibited higher physical health, which is in line with the previously mentioned studies.

The androgen receptor gene polymorphism was suggested to potentially alter the course of physical health. According to our results, the androgen receptor gene polymorphism does appear to influence the course of subjectively experienced physical health even after controlling for longitudinal T secretion. Surprisingly, however, previous findings emphasized the role of the androgen receptor gene polymorphism in physical health in the patient samples, but not in a general population sample (Schneider et al., 2010). Furthermore, it has been shown that the androgen receptor gene polymorphism can modulate androgen action in the target cells (Eendebak et al., 2016), thus suggesting a potential mechanism to explain its influence on physical health. This is relevant, for example, in the patient samples with potentially altered endocrine secretion patterns. In the present study, we were able to replicate the association with subjective physical health, thus further strengthening the link between the androgen receptor gene polymorphism and physical health.

The mean length for the androgen receptor gene polymorphism in the present study was 17.8, and thus below the generally reported mean of approximately 21. This can be explained by the applied capillary electrophoresis method. Other studies using the same method also reported lower repeats of approximately three base-pairs for the androgen receptor gene polymorphism (Mansfield et al., 1996; Westberg et al., 2001; Boorman et al., 2002). Capillary electrophoresis can potentially include a systematic error. Due to an extensive pairing in the CAG region, the probability that this region will melt completely during the analyses is decreased (Mansfield et al., 1996; Boorman et al., 2002). This can lead to an underestimation of the true repeat length when comparing fragment peaks with standard curves (Boorman et al., 2002).

Some limitations of the present study need to be mentioned. The first refers to the high dropout rate, which was mostly due to the *post hoc* planning of the follow-up. Second, potential conclusions that are drawn from this study only refer to healthy aging men. The additional investigation of a non-healthy group could be beneficial. Third, the methodological issues of the genetic analyses hinder comparisons of the androgen receptor gene polymorphism length with other study findings, although the sample itself was very homogeneous. Fourth, the internal consistency for the physical health questionnaire scale was rather low in the current sample. This may point to the need to validate the questionnaire in a healthy aging population. Finally, there are also some statistical constraints within the study: we used a two-time-point design, which could only incorporate random intercepts, whereas more time points would enable detailed random slope analyses (Curran et al., 2010).

Besides this, our study has several distinct strengths. The longitudinal design enabled the detailed investigation of intraindividual changes over time. Moreover, we used validated questionnaires and protocols for data collection. Applying strict inclusion criteria, we specifically investigated healthy aging men. Furthermore, we applied sophisticated statistical methods, respecting interindividual differences while focusing on intraindividual changes.

To sum up, we found that E2 influenced the course of psychological well-being, while T was a predictor of physical health. Furthermore, the androgen receptor gene polymorphism was associated with the course of self-reported physical health but not the course of psychological well-being. Since self-reported perceived health and actual health are closely related (Dainese et al., 2011), these results provide new insights into the role of distinct endocrine and genetic parameters for the maintenance of health throughout the aging process in men. However, the interactions of biological and psychological aspects in healthy aging men remain poorly understood and require further study.

## Data Availability Statement

The raw data supporting the conclusions of this article will be made available by the authors, without undue reservation, to any qualified researcher.

## Ethics Statement

The studies involving human participants were reviewed and approved by Ethics Committee of the Canton of Zurich. The patients/participants provided their written informed consent to participate in this study.

## Author Contributions

TL and UE designed the concept of the study. TL and AW organized and conducted the study and collected the data. TL wrote the first draft of the manuscript. UE, SF, and AW contributed with important intellectual content and edited subsequent versions of the manuscript.

## Conflict of Interest

The authors declare that the research was conducted in the absence of any commercial or financial relationships that could be construed as a potential conflict of interest.

## References

[B1] Adam E. K. Quinn M. E. Tavernier R. McQuillan M. T. Dahlke K. A. Gilbert K. E. (2017). Diurnal cortisol slopes and mental and physical health outcomes: a systematic review and meta-analysis. Psychoneuroendocrinology 83, 25–41. 10.1016/j.psyneuen.2017.05.018 28578301 PMC5568897

[B2] Barzilai N. Cuervo A. N. Austad S. (2018). Aging as a biological target for prevention and therapy. JAMA 320, 1321–1322. 10.1001/jama.2018.9562 30242337

[B3] Bates D. Mächler M. Bolker B. M. Walker S. C. (2015). Fitting linear mixed-effects models using lme4. J. Stat. Softw. 67:2015 10.18637/jss.v067.i01

[B4] Beard J. R. Bloom D. E. (2015). Towards a comprehensive public health response to population ageing. Lancet 385, 658–661. 10.1016/s0140-6736(14)61461-6 25468151 PMC4663973

[B5] Beard J. R. Officer A. De Carvalho I. A. Sadana R. Pot A. M. Michel J. P.. (2016). The world report on ageing and health: a policy framework for healthy ageing. Lancet 387, 2145–2154. 10.1016/S0140-6736(15)00516-4 26520231 PMC4848186

[B6] Beer T. M. Bland L. B. Bussiere J. R. Neiss M. B. Wersinger E. M. Garzotto M.. (2006). Testosterone loss and estradiol administration modify memory in men. J. Urol. 175, 130–135. 10.1016/S0022-5347(05)00049-2 16406889

[B7] Boorman D. W. Guo Y. Visvanathan K. Helzlsouer K. O’Brien T. G. (2002). Automated fragment analysis method for determining androgen receptor CAG repeat length. Biotechniques 33, 140–143. 10.2144/02331md01 12139238

[B8] Bourassa K. J. Memel M. Woolverton C. Sbarra D. A. (2017). Social participation predicts cognitive functioning in aging adults over time: comparisons with physical health, depression and physical activity. Aging Ment. Health 21, 133–146. 10.1080/13607863.2015.1081152 26327492

[B9] Bullinger M. Kirchberger I. (1998). SF-36: Fragebogen Zum Gesundheitszustand; Handanweisung. Göttingen: Hogrefe, Verlag für Psychologie.

[B10] Cannon W. B. (1929). Organization for physiological homeostasis. Physiol. Rev. 9, 399–431. 10.1152/physrev.1929.9.3.399

[B11] Chamberlain N. L. Driver E. D. Miesfeld R. L. (1994). The length and location of CAG trinucleotide repeats in the androgen receptor N-terminal domain affect transactivation function. Nucleic Acids Res. 22, 3181–3186. 10.1093/nar/22.15.3181 8065934 PMC310294

[B12] Chiappin S. Antonelli G. Gatti R. Elio F. (2007). Saliva specimen: a new laboratory tool for diagnostic and basic investigation. Clin. Chim. Acta 383, 30–40. 10.1016/j.cca.2007.04.011 17512510

[B13] Curran P. J. Obeidat K. Losardo D. (2010). Twelve frequently asked questions about growth curve modeling. J. Cogn. Dev. 11, 121–136. 10.1080/15248371003699969 21743795 PMC3131138

[B14] Dainese S. M. Allemand M. Ribeiro N. Bayram S. Martin M. Ehlert U. (2011). Protective factors in midlife. GeroPsych 24, 19–29. 10.1024/1662-9647/a000032

[B15] Demirev P. A. (2013). Dried blood spots: analysis and applications. Anal. Chem. 85, 779–789. 10.1021/ac303205m 23171435

[B16] Dennison E. Hindmarsh P. Fall C. Kellingray S. Barker D. Phillips D.. (1999). Profiles of endogenous circulating cortisol and bone mineral density in healthy elderly men. J. Clin. Endocrinol. Metab. 84, 3058–3063. 10.1210/jcem.84.9.5964 10487665

[B17] Denson T. F. Ronay R. von Hippel W. Schira M. M. (2013). Endogenous testosterone and cortisol modulate neural responses during induced anger control. Soc. Neurosci. 8, 165–177. 10.1080/17470919.2012.655425 22263640

[B18] Diener E. Lucas R. E. (1999). “Personality and subjective well-being,” in Well-Being: The Foundations of Hedonic Psychology, eds Diener E. Kahneman D. Schwarz N. (New York, NY: Russell Sage Foundation), 213–229.

[B19] Eendebak R. J. A. H. Huhtaniemi I. T. Pye S. R. Ahern T. W O’Neill T. Bartfai G.. (2016). The androgen receptor gene CAG repeat in relation to 4-year changes in androgen-sensitive endpoints in community-dwelling older european men. Eur. J. Endocrinol. 175, 583–593. 10.1530/eje-16-0447 27634944

[B20] Engel G. L. (1980). The clinical application of the biopsychosocial model. Am. J. Psychiatry 137, 535–544. 10.1176/ajp.137.5.535 7369396

[B21] Feldman H. A. Longcope C. Derby C. A. Johannes C. B. Araujo A. B. Coviello A. D.. (2002). Age trends in the level of serum testosterone and other hormones in middle-aged men: longitudinal results from the massachusetts male aging study. J. Clin. Endocrinol. Metab. 87, 589–598. 10.1210/jcem.87.2.8201 11836290

[B22] Fiacco S. Walther A. Ehlert U. (2018). Steroid secretion in healthy aging. Psychoneuroendocrinology 105, 64–78. 10.1016/j.psyneuen.2018.09.035 30314729

[B23] Fischer S. Ehlert U. Amiel Castro R. (2019a). Hormones of the hypothalamic-pituitary-gonadal (HPG) axis in male depressive disorders - a systematic review and meta-analysis. Front. Neuroendocrinol. 55:100792. 10.1016/j.yfrne.2019.100792 31557486

[B24] Fischer S. Obrist R. Ehlert U. (2019b). How and when to use dried blood spots in psychoneuroendocrinological research. Psychoneuroendocrinology 108:190–196. 10.1016/j.psyneuen.2019.06.011 31239081

[B25] Goncharov N. Katsya G. Dobracheva A. Nizhnik A. Kolesnikova G. Herbst V.. (2006). Diagnostic significance of free salivary testosterone measurement using a direct luminescence immunoassay in healthy men and in patients with disorders of androgenic status. Aging Male 9, 111–122. 10.1080/13685530600713060 16916746

[B26] Gong Y. Xiao H. Li C. Bai J. Cheng X. Jin M.. (2013). Elevated T/E 2 ratio is associated with an increased risk of cerebrovascular disease in elderly men. PLoS One 8:e61598. 10.1371/journal.pone.0061598 23637864 PMC3634802

[B27] Gordon E. H. Hubbard R. E. (2018). Physiological basis for sex differences in frailty. Curr. Opin. Physiol. 6, 10–15. 10.1016/j.cophys.2018.02.013

[B28] Gupta D. Morley J. E. (2014). Hypothalamic-pituitary-adrenal (HPA) axis and aging. Compr. Physiol. 4, 1495–1510. 10.1002/cphy.c130049 25428852

[B29] Guthrie B. Boyd C. M. (2018). Clinical guidelines in the context of aging and multimorbidity. Public Policy Aging Rep. 28, 143–149. 10.1093/ppar/pry038

[B30] Han K. Lee Y. Gu J. Oh H. Han J. Kim K. (2015). Psychosocial factors for influencing healthy aging in adults in Korea. Health Qual. Life Outcomes 13:31. 10.1186/s12955-015-0225-5 25879942 PMC4367838

[B31] Hansen T. Slagsvold B. (2012). The age and subjective well-being paradox revisited: a multidimensional perspective. Norsk Epidemiol. 22, 187–195. 10.5324/nje.v22i2.1565

[B32] Hsu B. Cumming R. G. Naganathan V. Blyth F. M. Le Couteur D. G. Hirani V.. (2016). Temporal changes in androgens and estrogens are associated with all-cause and cause-specific mortality in older men. J. Clin. Endocrinol. Metab. 101, 2201–2210. 10.1210/jc.2016-1025 26963953

[B33] Huhtaniemi I. T. Pye S. R. Limer K. L. Thomson W. O’Neill T. W. Platt H.. (2009). Increased estrogen rather than decreased androgen action is associated with longer androgen receptor CAG repeats. J. Clin. Endocrinol. Metab. 94, 277–284. 10.1210/jc.2008-0848 18840639

[B34] Huisman M. Poppelaars J. van der Horst M. Beekman A. T. F. Brug J. van Tilburg T. G.. (2011). Cohort profile: the longitudinal aging study amsterdam. Int. J. Epidemiol. 40, 868–876. 10.1093/ije/dyQ239 21216744

[B35] Joseph D. N. Whirledge S. (2017). Stress and the HPA axis: balancing homeostasis and fertility. Int. J. Mol. Sci. 18:E2224. 10.3390/ijms18102224 29064426 PMC5666903

[B36] Karlamangla A. S. Friedman E. M. Seeman T. E. Stawksi R. S. Almeida D. M. (2013). Daytime trajectories of cortisol: demographic and socioeconomic differences-findings from the national study of daily experiences. Psychoneuroendocrinology 38, 2585–2597. 10.1016/j.psyneuen.2013.06.010 23831263 PMC3812359

[B37] Krithivas K. Yurgalevitch S. M. Mohr B. A. Wilcox C. J. Batter S. J. Brown M.. (1999). Evidence that the CAG repeat in the androgen receptor gene is associated with the age-related decline in serum androgen levels in men. J. Endocrinol. 162, 137–142. 10.1677/joe.0.1620137 10396030

[B38] Kunzmann U. Little T. D. Smith J. (2000). Is age-related stability of subjective well-being a paradox? Cross-sectional and longitudinal evidence from the Berlin aging study. Psychol. Aging 15, 511–526. 10.1037/0882-7974.15.3.511 11014714

[B39] Lacker T. J. Walther A. Ehlert U. (2019). Age-related alterations in endocrine markers do not match changes in psychosocial measures - findings from the men’s health 40+ longitudinal study. Psychoneuroendocrinology 107, 17–18. 10.1016/j.psyneuen.2019.07.048 PMC7278106 32456528

[B40] Lowsky D. J. Olshansky S. J. Bhattacharya J. Goldman D. P. (2013). Heterogeneity in healthy aging. J. Gerontol. A Biol. Sci. Med. Sci. 69, 640–649. 10.1093/gerona/glt162 24249734 PMC4022100

[B41] Mansfield E. S. Vainer M. Enad S. Barker D. L. Harris D. Rappaport E.. (1996). Sensitivity, reproducibility, and accuracy in short tandem repeat genotyping using capillary array electrophoresis. Genome Res. 6, 893–903. 10.1101/gr.6.9.893 8889558

[B42] Menke A. Guallar E. Rohrmann S. Nelson W. G. Rifai N. Kanarek N.. (2010). Sex steroid hormone concentrations and risk of death in US men. Am. J. Epidemiol. 171, 583–592. 10.1093/aje/kwp415 20083549 PMC6596446

[B43] Monteagudo P. T. Falcão A. A. Verreschi I. T. N. Zanella M. T. (2016). The imbalance of sex-hormones related to depressive symptoms in obese men. Aging Male 19, 20–26. 10.3109/13685538.2015.1084500 26488864

[B44] Murphy S. L. Xu J. Kochanek K. D. Curtin S. C. Arias E. (2017). Deaths: final data for 2015. Natl. Vital Stat. Rep. 66, 1–75. Available online at: https://stacks.cdc.gov/view/cdc/50011. 29235985

[B45] Nelson K. A. Witte J. S. (2002). Androgen receptor CAG repeats and prostate cancer. Am. J. Epidemiol. 155, 883–890. 10.1093/aje/155.10.883 11994226

[B46] Rajender S. Pandu G. Sharma J. D. Gandhi K. P. C. Singh L. Thangaraj K. (2008). Reduced CAG repeats length in androgen receptor gene is associated with violent criminal behavior. Int. J. Legal Med. 122, 367–372. 10.1007/s00414-008-0225-7 18365230

[B47] Rector B. J. L. Friedman E. M. (2018). “Hormones and well-being,” in Handbook of Well-Being, eds Diener E. Oishi S. Tay L. (Salt Lake City, UT: DEF Publishers), 1–16.

[B48] Rockwood K. (2019). Frailty and aging medicine. Aging Med. 2, 4–6. 10.1002/agm2.12060 31942506 PMC6880699

[B49] Samaras N. Frangos E. Forster A. Lang P. O. Samaras D. (2012). Andropause: a review of the definition and treatment. Eur. Geriatr Med. 3, 368–373. 10.1016/j.eurger.2012.08.007

[B50] Schneider G. Nienhaus K. Gromoll J. Heuft G. Nieschlag E. Zitzmann M. (2010). Aging males’ symptoms in relation to the genetically determined androgen receptor CAG polymorphism, sex hormone levels and sample membership. Psychoneuroendocrinology 35, 578–587. 10.1016/j.psyneuen.2009.09.008 19804943

[B51] Smith G. D. Ben-Shlomo Y. Beswick A. Yarnell J. Lightman S. Elwood P. (2005). Cortisol, testosterone and coronary heart disease: prospective evidence from the caerphilly study. Circulation 112, 332–340. 10.1161/CIRCULATIONAHA.104.489088 16009799

[B52] Snyder P. J. Kopperdahl D. L. Stephens-Shields A. J. Ellenberg S. S. Cauley J. A. Ensrud K. E.. (2017). Effect of testosterone treatment on volumetric bone density and strength in older men with low testosterone: a controlled clinical trial. JAMA Intern. Med. 177, 471–479. 10.1001/jamainternmed.2016.9539 28241231 PMC5433755

[B53] Sollberger S. Ehlert U. (2016). How to use and interpret hormone ratios. Psychoneuroendocrinology 63, 385-397. 10.1016/j.psyneuen.2015.09.031 26521052

[B54] Strickhouser J. E. Zell E. Krizan Z. (2017). Does personality predict health and well-being? A metasynthesis. Health Psychol. 36, 797-810. 10.1037/hea0000475 28277701

[B55] Terburg D. Morgan B. van Honk J. (2009). The testosterone-cortisol ratio: a hormonal marker for proneness to social aggression. Int. J. Law Psychiatry 32, 216–223. 10.1016/j.ijlp.2009.04.008 19446881

[B56] Tirabassi G. Cignarelli A. Perrini S. delli Muti N. Furlani G. Gallo M.. (2015). Influence of CAG repeat polymorphism on the targets of testosterone action. Int. J. Endocrinol. 2015:298107. 10.1155/2015/298107 26421011 PMC4572434

[B58] Vermeulen A. (2000). Andropause. Maturitas 34, 5–15. 10.1016/s0378-5122(99)00075-4 10687877

[B59] Vermeulen A. Kaufman J. M. Goemaere S. Van Pottelberg I. (2002). Estradiol in elderly men. Aging Male 5, 98-102. 10.1080/tam.5.2.98.102 12198740

[B60] Viau V. (2002). Functional cross-talk between the hypothalamic-pituitary-gonadal and -Adrenal axes. J. Neuroendocrinol. 14, 506–513. 10.1046/j.1365-2826.2002.00798.x 12047726

[B61] Wade D. T. Halligan P. W. (2017). The biopsychosocial model of illness: a model whose time has come. Clin. Rehabil. 31, 995–1004. 10.1177/0269215517709890 28730890

[B62] Wallerand H. Rémy-Martin A. Chabannes E. Bermont L. Adessi G. L. Bittard H. (2001). Relationship between expansion of the CAG repeat in exon 1 of the androgen receptor gene and idiopathic male infertility. Fertil. Steril. 76, 769–774. 10.1016/s0015-0282(01)01987-2 11591412

[B63] Walther A. Ehlert U. (2015). Steroid secretion and psychological well-being in men 40+. Neurobiology of Men’s Mental Health. Available online at: http://ovidsp.ovid.com/ovidweb.cgi?T=JS…PAGE=reference…D=psyc12…NEWS=N…AN=2015-46819-024. Accessed September 17, 2019.

[B64] Walther A. Breidenstein J. Miller R. (2019). Association of testosterone treatment with alleviation of depressive symptoms in men: a systematic review and meta-analysis. JAMA Psychiatry 76, 31–40. 10.1001/jamapsychiatry.2018.2734 30427999 PMC6583468

[B65] Walther A. Philipp M. Lozza N. Ehlert U. (2016). The rate of change in declining steroid hormones: a new parameter of healthy aging in men? Oncotarget 7, 60844-60857. 10.18632/oncotarget.11752 27589836 PMC5308620

[B66] Wang L. Zhao X. Chen J. Guo X. Liang X. Yi D.. (2013). Biological indicators of sub-optimal health status. J. Tradit. Chin. Med. 33, 647–650. 10.1016/s0254-6272(14)60036-4 24660590

[B67] Weiss A. Bates T. C. Luciano M. (2008). Happiness is a Personal(ity) thing: the genetics of personality and well-being in a representative sample. Psychol. Sci. 19, 205–210. 10.1111/j.1467-9280.2008.02068.x 18315789

[B68] Westberg L. Baghaei F. Rosmond R. Hellstrand M. Landén M. Jansson M.. (2001). Polymorphisms of the androgen receptor gene and the estrogen receptor β gene are associated with androgen levels in women. J. Clin. Endocrinol. Metab. 86, 2562–2568. 10.1210/jcem.86.6.7614 11397855

[B69] Westberg L. Henningsson S. Landén M. Annerbrink K. Melke J. Nilson S.. (2009). Influence of androgen receptor repeat polymorphisms on personality traits in men. J. Psychiatry Neurosci. 34, 205–213. 19448851 PMC2674974

[B70] World Health Organization (2015a). World Health Statistics 2015. Luxemburg: World Health Organization.

[B71] World Health Organization (2015b). World Report on Ageing and Health. Luxemburg: World Health Organization.

[B72] World Health Organization (2017). 10 priorities towards a decade of healthy ageing, 1–20. Available online at: https://www.who.int/ageing/WHO-ALC-10-priorities.pdf. Accessed September 17, 2019.

[B73] Zhou J. N. Swaab D. F. (1999). Activation and degeneration during aging: a morphometric study of the human hypothalamus. Microsc. Res. Tech. 44, 36–48. 10.1002/(SICI)1097-0029(19990101)44:1<36::AID-JEMT5>3.0.CO;2-F 9915562

