# Supplementary material for: The Relation Between Steroid Secretion Patterns and the Androgen Receptor Gene Polymorphism on Physical Health and Psychological Well-Being—Longitudinal Findings From the Men’s Health 40+ Study
Source: Front Hum Neurosci. 2020 Feb 14;14:43. doi: 10.3389/fnhum.2020.00043 (PMC7033643; doi:10.3389/fnhum.2020.00043)
Supplement: Supplementary file 1 [file Table_1.docx]

**Supplementary Material**

Table S1. Zero-order correlations

| **Matrix of zero-order correlations** | | | | | | | | | | | | | | |
| --- | --- | --- | --- | --- | --- | --- | --- | --- | --- | --- | --- | --- | --- | --- |
|  | 1 | 2 | 3 | 4 | 5 | 6 | 7 | 8 | 9 | 10 | 11 | 12 | 13 | 14 |
| 1. CAG repeat |  |  |  |  |  |  |  |  |  |  |  |  |  |  |
| 2. TCr (T1) | -.165 |  |  |  |  |  |  |  |  |  |  |  |  |  |
| 3. TCr (T2) | -.293^**^ | .070 |  |  |  |  |  |  |  |  |  |  |  |  |
| 4. TE2r (T1) | -.032 | .045 | .143 |  |  |  |  |  |  |  |  |  |  |  |
| 5. TE2r (T2) | -.181^†^ | -.115 | .517^***^ | .348^***^ |  |  |  |  |  |  |  |  |  |  |
| 6. C (T1) | .177^†^ | -.511^***^ | -.043 | -.024 | .237^*^ |  |  |  |  |  |  |  |  |  |
| 7. C (T2) | .095 | -.090 | -.273^*^ | .066 | -.049 | .078 |  |  |  |  |  |  |  |  |
| 8. E2 (T1) | .134 | .129 | .026 | -.640^***^ | -.268^*^ | .052 | -.089 |  |  |  |  |  |  |  |
| 9. E2 (T2) | .211^*^ | .067 | .158 | -.265^*^ | -.347^***^ | -.106 | .033 | .370^***^ |  |  |  |  |  |  |
| 10. T (T1) | .088 | .316^**^ | .081 | .074 | -.060 | .153 | -.054 | .456 | .150 |  |  |  |  |  |
| 11. T (T2) | .024 | .066 | .588^***^ | .067 | .444^***^ | .183^†^ | -011 | .187^†^ | .438^***^ | .274^**^ |  |  |  |  |
| 12. Phys. health (T1) | .164 | -.019 | -.177 | .238^*^ | -.009 | .091 | .236^*^ | .021 | -.029 | .307^**^ | .146 |  |  |  |
| 13. Phys. health (T2) | -.065 | .077 | -.104 | .157 | .010 | .086 | .194^†^ | .045 | -.016 | .274^**^ | .183^†^ | .627^***^ |  |  |
| 14. Psych. wellb. (T1) | .046 | .188^†^ | .058 | -.238^*^ | -.049 | -.023 | .187^†^ | .171 | .176 | .028 | .102 | .225^*^ | .227^*^ |  |
| 15. Psych. wellb. (T2) | -.004 | .113 | .000 | -.043 | -.032 | -.043 | .207^†^ | .068 | .131 | .163 | .155 | .358^**^ | .386^***^ | .618^***^ |

Note. † = p<.10, * = p<.05, ** = p<.01, *** = p<.001. TCr = Testosterone/Cortisol ratio, TE2r = Testosterone/Estradiol ratio, C = Cortisol, E2 = Estradiol, T = Testosterone, Phys. health = Physical health, Psych. well-being = Psychological well-being.

| Table S2. Partial correlations  **Matrix of partial correlations** | | | | | | | | | | | | | | |
| --- | --- | --- | --- | --- | --- | --- | --- | --- | --- | --- | --- | --- | --- | --- |
|  | 1 | 2 | 3 | 4 | 5 | 6 | 7 | 8 | 9 | 10 | 11 | 12 | 13 | 14 |
| 1. CAG repeat |  |  |  |  |  |  |  |  |  |  |  |  |  |  |
| 2. TCr (T1) | -.155 |  |  |  |  |  |  |  |  |  |  |  |  |  |
| 3. TCr (T2) | -.353^**^ | .066 |  |  |  |  |  |  |  |  |  |  |  |  |
| 4. TE2r (T1) | .037 | .030 | .168 |  |  |  |  |  |  |  |  |  |  |  |
| 5. TE2r (T2) | -.175 | -.095 | .554^***^ | .340^**^ |  |  |  |  |  |  |  |  |  |  |
| 6. C (T1) | .250^*^ | -.095^***^ | -.040 | -.049 | .262^*^ |  |  |  |  |  |  |  |  |  |
| 7. C (T2) | .124 | -.087 | -.286^*^ | .047 | -.113 | .170 |  |  |  |  |  |  |  |  |
| 8. E2 (T1) | .074 | .142 | .029 | -.630^***^ | -.227^*^ | .063 | -.044 |  |  |  |  |  |  |  |
| 9. E2 (T2) | .188 | .056 | .125 | -.256* | -.361^**^ | -.073 | .059 | .386^**^ |  |  |  |  |  |  |
| 10. T (T1) | .133 | .340^*^ | .115 | .066 | -.007 | .059 | .080 | .468^***^ | .223^†^ |  |  |  |  |  |
| 11. T (T2) | .011 | .091 | .612^***^ | .090 | .446^***^ | .155 | .023 | .194^†^ | .482^***^ | .284^**^ |  |  |  |  |
| 12. Phys. health (T1) | .258^*^ | -.015 | -.100 | .218^†^ | -.026 | .018 | .229* | .059 | .056 | .281^*^ | .150 |  |  |  |
| 13. Phys. health (T2) | -.002 | .103 | .017 | .167 | .006 | .016 | .135 | .074 | .091 | .306^**^ | .192^†^ | .573^***^ |  |  |
| 14. Psych. wellb. (T1) | -.013 | .243^*^ | .009 | -.173 | -.021 | .032 | .189 | .193^†^ | .164 | .202^†^ | .190 | .330^**^ | .329^**^ |  |
| 15. Psych. wellb. (T2) | -.033 | .131 | .082 | -.051 | -.018 | -.009 | .151 | .054 | .172 | .225^†^ | .210^†^ | .355^**^ | .388^**^ | .666^***^ |
| Note. † = p<.10, * = p<.05, ** = p<.01, *** = p<.001, partial correlations controlled for education, income, fat-mass, physical activity and age. TCr = Testosterone/Cortisol ratio, TE2r = Testosterone/Estradiol ratio, C = Cortisol, E2 = Estradiol, T = Testosterone, Phys. health = Physical health, Psych. well-being = Psychological well-being. | | | | | | | | | | | | | | |
